# High-flux, moderate-bandwidth, high-energy X-rays from lapped Si(111) crystals in a double-crystal monochromator

**DOI:** 10.1107/S1600577526004960

**Published:** 2026-06-16

**Authors:** Hiroshi Yamazaki, Kazuhiko Tahara, Yasuhiro Shimizu, Haruhiko Ohashi

**Affiliations:** a Japan Synchrotron Radiation Research Institute 1-1-1 Kouto Sayo Hyogo679-5198 Japan; b RIKEN SPring-8 Center, 1-1-1 Kouto, Sayo, Hyogo679-5148, Japan; Brazilian Synchrotron Light Laboratory, Brazil

**Keywords:** lapped Si(111) double-crystal monochromator, high-energy X-ray beams, high flux, moderate bandwidth, flux enhancement

## Abstract

Lapping the surfaces of Si(111) crystals in a double-crystal monochromator significantly increases the intensity of high-energy X-ray beams. Systematic characterization using 100 keV X-rays shows that the total flux increases by factors of 7–11 relative to polished crystals. The resulting energy bandwidth (0.12–0.21%) remains substantially narrower than that of multilayer optics. These characteristics provide a useful compromise between spectral selectivity and beam intensity, enabling practical X-ray measurements that are difficult to achieve with either perfect-crystal monochromators or multilayer optics alone. Demonstrations of imaging, powder diffraction, and incoherent micro-focusing further highlight the practical utility of the resulting beams in applications where high photon statistics are prioritized. These results demonstrate that lapped Si crystals provide a robust and cost-effective approach to generating high-flux, moderate-bandwidth high-energy X-ray beams.

## Abstract 2

Lapped Si(111) crystals in a double-crystal monochromator provide high-flux, spectrally pure, and moderate-bandwidth X-ray beams at 100 keV, offering a robust and cost-effective alternative to perfect-crystal and multilayer optics.

## 1. Introduction

High-energy X-rays are increasingly utilized in synchrotron-based experiments that require penetration through bulk materials. In diffraction-based measurements, high photon energies enable access to deeply buried regions in thick samples, allowing structural information to be obtained from within bulk materials.

However, the efficient use of high-energy X-rays remains challenging. Conventional perfect-crystal double-crystal monochromators (DCMs) provide excellent spectral selectivity, but their extremely narrow angular acceptance leads to a significant loss of delivered flux from realistic synchrotron beams. On the other hand, multilayer optics offer substantially higher flux, but at the expense of much broader energy bandwidth and reduced spectral resolution (Koyama *et al.*, 2022 ▸; Yumoto *et al.*, 2025 ▸). This situation motivates the development of X-ray optics that provide a balance between spectral selectivity and photon flux.

One approach is to use bent Laue monochromators (Suortti *et al.*, 1992 ▸; Suortti & Schulze, 1995 ▸), in which the Rowland geometry effectively facilitates capturing source divergences, offering high-flux X-rays. This scheme can precisely control beam wavefronts through sophisticated bending mechanisms and precise alignment. In particular, the first demonstration of cryogenically cooled Laue DCMs (Shastri *et al.*, 2002 ▸) has led to adoption of the concept at numerous facilities worldwide (ESRF, PETRA, DIAMOND, ANSTO, CHESS, NSLS, and HEPS). However, many diffraction-based measurements do not strictly require a pristine wavefront.

A simpler, alternative approach to increase integrated reflectivity is to introduce imperfections directly into crystals. The increase can be understood as arising from a combination of reduced extinction effects and a broadened distribution of local lattice orientations (Darwin, 1922 ▸; Hamilton, 1957 ▸; Zachariasen, 1967 ▸). Although an intrinsic peak reflectivity reduces compared with the perfect-crystal case, dispersed lattice orientations allow access to a wider range of incident angles and energies. Enhanced reflectivity from deliberately processed imperfect crystals has been demonstrated, for example in compressed Ge single crystals used as neutron monochromators (Barrett *et al.*, 1963 ▸). For X-rays, annealed Czochralski grown Si crystals including internal imperfections have been investigated for monochromators, where net gains of integrated reflectivity were shown to increase as photon energy increases (Schneider *et al.*, 1988 ▸; Joksch *et al.*, 1993 ▸). Shiwaku *et al.* (1991 ▸, 1992 ▸) also reported enhanced reflectivity from surface-lapped Si crystals. Despite these early observations, imperfect crystals have found only limited application in DCMs, because the broadened angular acceptance was expected to degrade beam collimation and to affect the efficiency of non-dispersive double-crystal operation.

Recently, this assumption has begun to be reconsidered. Hoshino & Uesugi (2017 ▸) conducted test operations of surface-lapped Si crystals in a DCM at the 200 m-long beamline BL20B2 at SPring-8 and demonstrated imaging experiments, albeit under conditions involving long-distance beam propagation. However, the detailed beam properties were not characterized, and the applicability to other experimental modalities remains unclear.

In this study, the performance of lapped Si(111) crystals in a DCM is experimentally evaluated for high-energy X-ray applications, exemplified here by 100 keV photons. The beam properties are systematically characterized in terms of flux, energy bandwidth, angular divergence, and beam uniformity, and are compared with those obtained using surface-polished crystals and a double multilayer monochromator (DMM). Representative imaging, diffraction, and micro-focusing experiments are also performed to demonstrate the practical applicability of the resulting beams. While the present work focuses on these practical evaluations, a more detailed analysis of the wavelength–angle distributions will be addressed in a separate study (Yamazaki *et al.*, 2026 ▸).

## 2. Experimental

### 2.1. Crystal fabrication

Lapping introduces microscopic imperfections into the reflecting crystal surface. To obtain a homogeneous beam, the degree of surface damage must be uniform across the illuminated footprint. To ensure such precise control over surface roughness, the crystals were prepared in the following manner.

Initially, several silicon blocks with dimensions of 140 mm × 25 mm × 35 mm were prepared from non-dope floating-zone Si ingots. For each block, the reflecting plane was the (111) plane corresponding to the 140 mm × 25 mm surface. The two 140 mm × 35 mm faces were oriented as 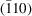 planes, to which the lateral clamping forces would be applied. Subsurface damage was removed entirely by chemical etching, after which the three aforementioned faces were finished using mechanochemical polishing. Two of these polished blocks were reserved as a reference, hereafter referred to as ‘polished crystals’.

The reflecting planes were then lapped using alumina abrasives in water, with a glass plate serving as the lapping tool. The process was performed by hand in a circular motion while maintaining a constant crystal orientation, until a uniform finish was obtained. Although the lapping was not controlled on the time-controlled basis, the net lapping time (excluding inspection time) was approximately half an hour per crystal. Four pairs of lapped crystals were prepared, where the two crystals in each pair were finished using the same abrasive grit. The grit numbers used were #400 (median particle diameter 30–34 µm; surface roughness *R*_*q*_ = 910 nm), #800 (14–18 µm; 430 nm), #1200 (9.5–13 µm; 400 nm), and #2000 (6.7–8.5 µm; 240 nm). Because the etching of lapped surfaces cannot be controlled with sufficient precision, no additional etching was performed after the lapping process.

### 2.2. Optical configuration

**Figure 1:**
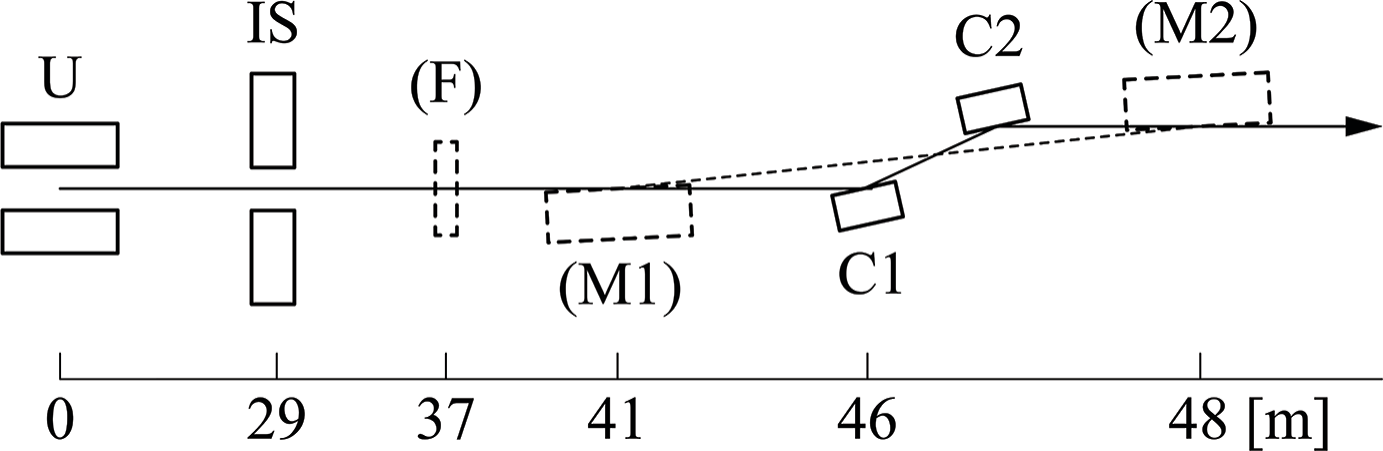
Optical layout of the beamline. Distances from the undulator (U) are indicated in metres. IS: incident slit; C1 and C2: DCM crystals. The multilayer mirrors (M1 and M2) were used as a reference together with a low-energy-cut filter (F) to suppress the total reflection from the mirrors. The DCM and DMM were operated alternately.

The experiments were performed at beamline BL05XU of SPring-8. Fig. 1 ▸ illustrates the beamline optics. The beamline (Yumoto *et al.*, 2020 ▸) was formerly equipped with an in-vacuum planar undulator comprising 93 magnet pairs with a period of 32 mm, which produced the maximum intensity of 100 keV radiation at the 22nd harmonic for a magnet gap of 8.42 mm. The beam size was defined by an incident slit with an opening of 0.56 mm (vertical) and 1.86 mm (horizontal), located 29 m downstream from the undulator.

The DCM was configured in a vertical scattering geometry. The first crystals were installed 46 m from the undulator on a high-precision goniometer with an angular step size of approximately 100 nrad, using a tenfold stepping-motor drive. A rotary encoder provided a finer resolution of about 8 nrad, enabling precise monitoring of the angular positions. Liquid nitrogen was circulated at 8.4 L min^−1^ through vacuum-insulated transfer lines to cool the crystals. The crystal holder was designed to mount two crystals (*e.g.* #400 and #800) side by side, which were clamped laterally using cryogenically cooled copper plates. For the measurements conducted at 100 keV, which was chosen to evaluate the performance in the high-energy region, the Bragg angle was 1.13° (19.8 mrad).

The corresponding second crystals, finished with the same surface roughness as the first, were mounted on a similar goniometer 68 cm downstream of the first crystals. Because the thermal load was significantly reduced after the first reflection, the second crystals were operated at room temperature without active cooling.

The beamline is also equipped with a DMM providing a bandwidth of 1.0% (Yumoto *et al.*, 2025 ▸). For comparison, selected measurements were performed using the DMM with the DCM retracted. Because the glancing angle at 100 keV is 1.91 mrad, the effective mirror length required to intercept the same beam cross section is approximately ten times that of the Si crystals. Since the DMM totally reflects low-energy X-rays below 40 keV, a low-energy-cut filter—consisting of 1.2 mm diamond, 1.4 mm SiC, 8.0 mm Si, and 0.1 mm Mo—was inserted. The filter was removed when using the DCMs.

**Figure 2:**
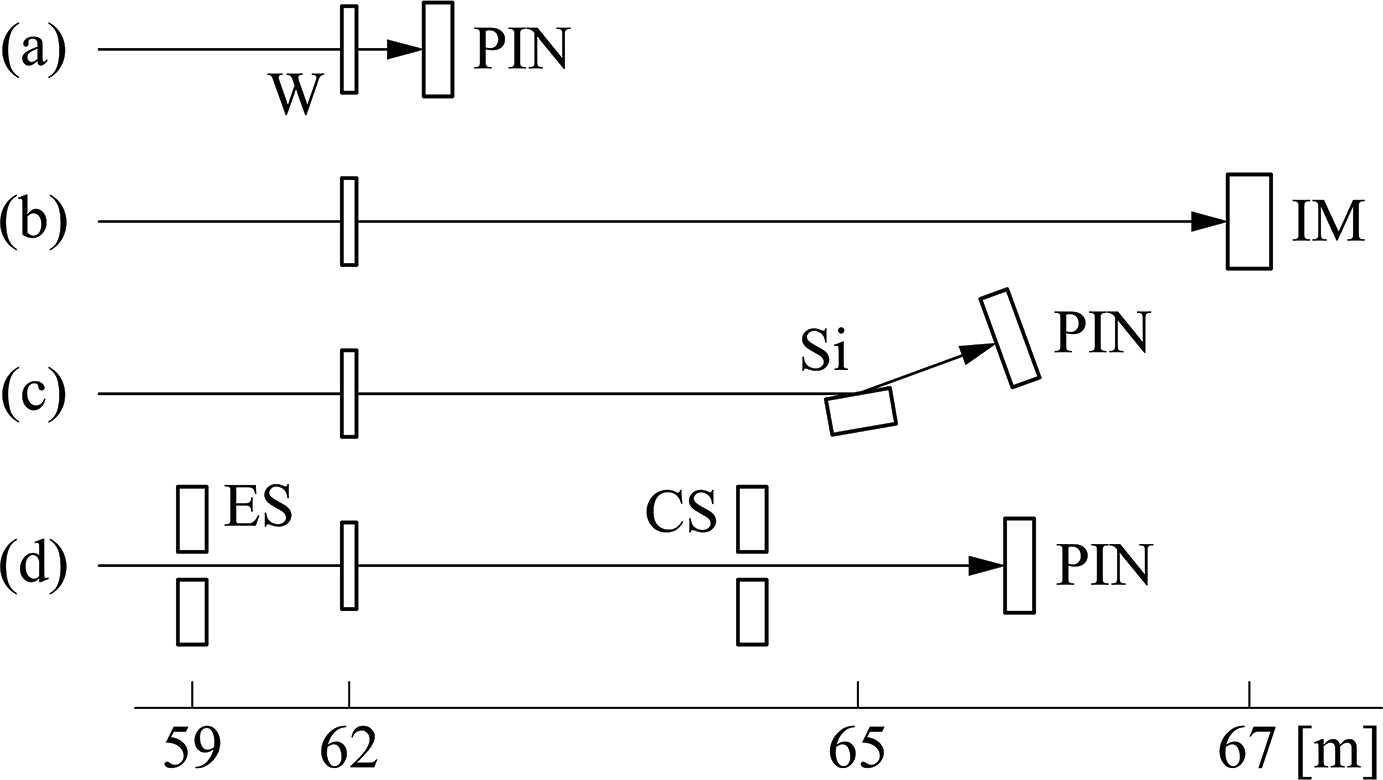
Experimental setups for beam characterization. (*a*) Total flux measurement using a silicon PIN photodiode placed downstream of the exit window (W). (*b*) Beam imaging using a beam-image monitor (IM). (*c*) θ–2θ scan using a Si(111) analyzer crystal to evaluate the wavelength–angle distribution. (*d*) Flux density measurement using the exit slit (ES) and a downstream clean-up slit (CS).

After passing through the exit slit located 59 m from the undulator, the monochromated beams were delivered to the experimental station at 62 m via Be and Si_3_N_4_ windows (Figs. 2 ▸ and 3 ▸).

### 2.3. Evaluation of beam quality

The beam intensity was measured using a silicon PIN photodiode [S14537-320, Hamamatsu Photonics KK, packaged by Ohyo Koken Kogyo Co., Ltd.; Fig. 2 ▸(*a*)]. The photodiode signals were recorded at 1 kHz with an integration time of 1 ms. With the exit slit fully opened, the first-crystal goniometer was rotated continuously at approximately 50 µrad s^−1^ for the lapped pairs and at about 100 nrad s^−1^ for the polished pair. Encoder counts were synchronously recorded at 1 kHz to obtain rocking curves. The peak intensity of each rocking curve was taken as the total beam flux.

Two-dimensional beam profiles were recorded using a beam-image monitor (Kameshima *et al.*, 2019 ▸) positioned 67 m from the undulator [Fig. 2 ▸(*b*)]. A LuAG:Ce scintillator with a thickness of 10 µm was used to obtain higher gain instead of the 5 µm described in the article.

To characterize the wavelength–angle distribution, a polished Si(111) analyzer crystal was mounted on the θ stage of a θ–2θ diffractometer [Fig. 2 ▸(*c*)]. The resulting θ–2θ rocking curves were broadened by both the wavelength spread and angular divergence of the incident beam, with both effects varying according to the Bragg angles of the analyzer crystal. From the rocking-curve profiles measured for reflections 111 through 777 and 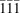 through 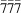, wavelength–angle distributions were reconstructed using an algebraic computed-tomography technique; further methodological details are provided elsewhere (Yamazaki *et al.*, 2026 ▸).

Flux densities were measured by defining a 0.2 mm × 0.2 mm aperture at the beam center. Because the edges of the exit slit produced parasitic reflections, an additional clean-up slit was placed downstream to eliminate these unwanted components [Fig. 2 ▸(*d*)].

### 2.4. Demonstrative applications

**Figure 3:**
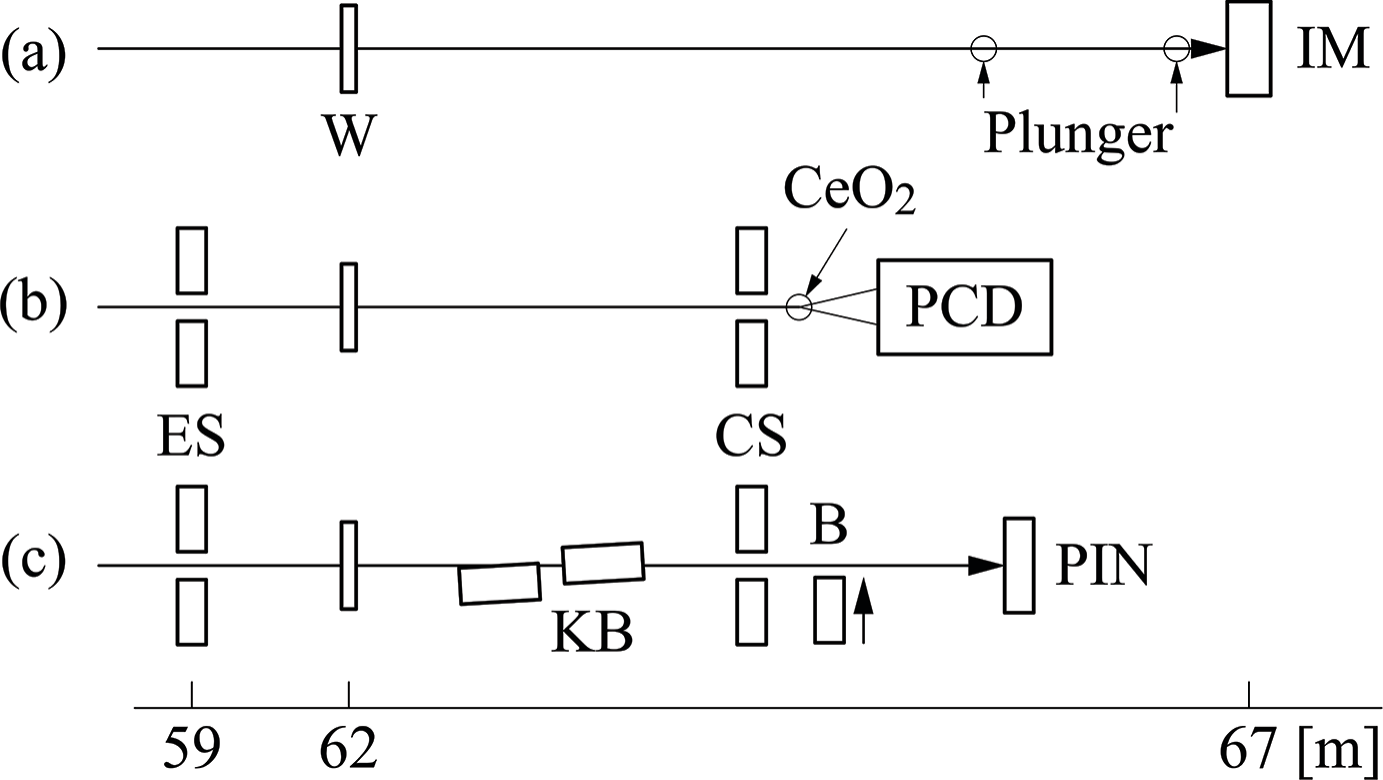
Experimental setups for application demonstrations. (*a*) Transmission imaging of a spring-ball plunger. (*b*) Powder diffraction measurement of CeO_2_ using a photon-counting detector (PCD). (*c*) Focusing with multilayer-coated KB mirrors and knife-edge scans using a tungsten blade (B).

Transmission images of a 2 mm-diameter spring-ball plunger were recorded using the beam image monitor, with the plunger positioned 6 mm and 2 m upstream from the image monitor [Fig. 3 ▸(*a*)].

Powder diffraction patterns of CeO_2_ were measured using a photon-counting detector (Eiger2 1M, CdTe, Dectris) positioned 13 cm downstream of the sample [Fig. 3 ▸(*b*)]. A 0.2 mm × 0.2 mm beam defined by the exit and clean-up slits was used.

To demonstrate focusing performance, a multilayer-coated Kirkpatrick–Baez (KB) mirror system, originally developed for focusing the 100 keV DMM beam (Koyama *et al.*, 2024 ▸), was employed. The mirrors were designed with demagnification ratios of 91 (vertical) and 135 (horizontal). The vertical size of the light source at the 100 keV component was calculated at approximately 15 µm (full width at half-maximum, FWHM) from the synchrotron radiation calculator *SPECTRA*, Version 12 (Tanaka & Kitamura, 2001 ▸; Tanaka, 2021 ▸). Based on these values, the system is geometrically capable of focusing to 0.16 µm in the vertical direction, assuming no errors. The exit slit was opened to 0.30 mm (vertical) and 0.28 mm (horizontal) to ensure that the beam footprint remained within the central 90% of the mirror lengths. Stray light was suppressed using the clean-up slit. The focal spot size was evaluated by performing knife-edge scans with a tungsten blade [Fig. 3 ▸(*c*)].

## 3. Results

### 3.1. Beam performance

Table 1 ▸ summarizes the main beam parameters obtained using the polished and lapped crystals, as well as the DMM and a low-energy-cut filter.

#### 3.1.1. Total flux and rocking-curve width

**Figure 4:**
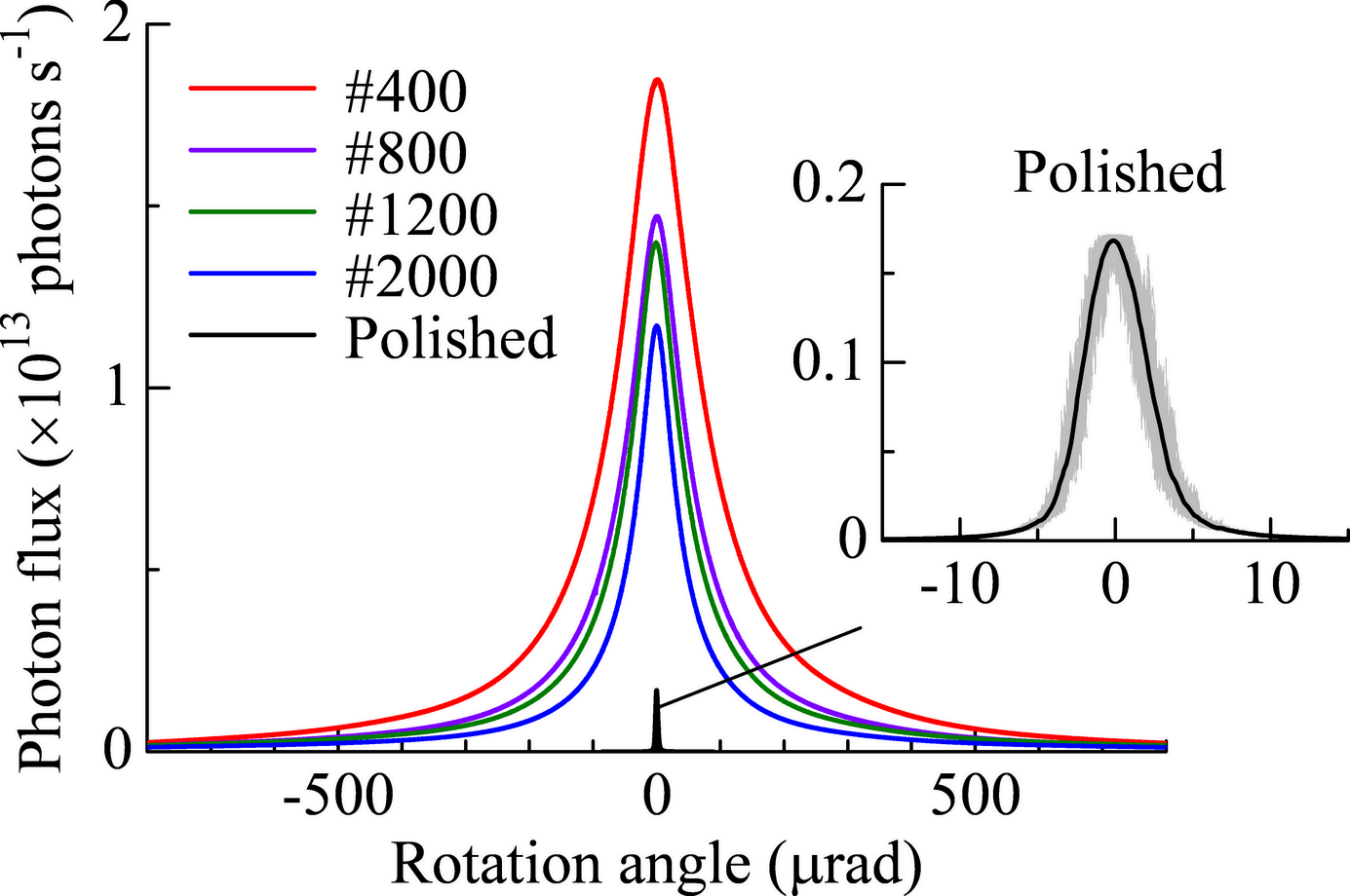
Rocking-curve profiles obtained by rotating the first-crystal stage. Increasing surface roughness (lower grit number) results in higher peak intensity and broader rocking-curve width. The inset shows the measured (gray) and averaged (black) rocking curves for the polished crystals.

Fig. 4 ▸ shows the rocking curves obtained by rotating the first-crystal stage. As the surface roughness increased (corresponding to smaller abrasive grit numbers), both the peak intensity and the rocking-curve width increased. The inset displays the rocking curve for the polished crystals, together with the averaged profile.

**Figure 5:**
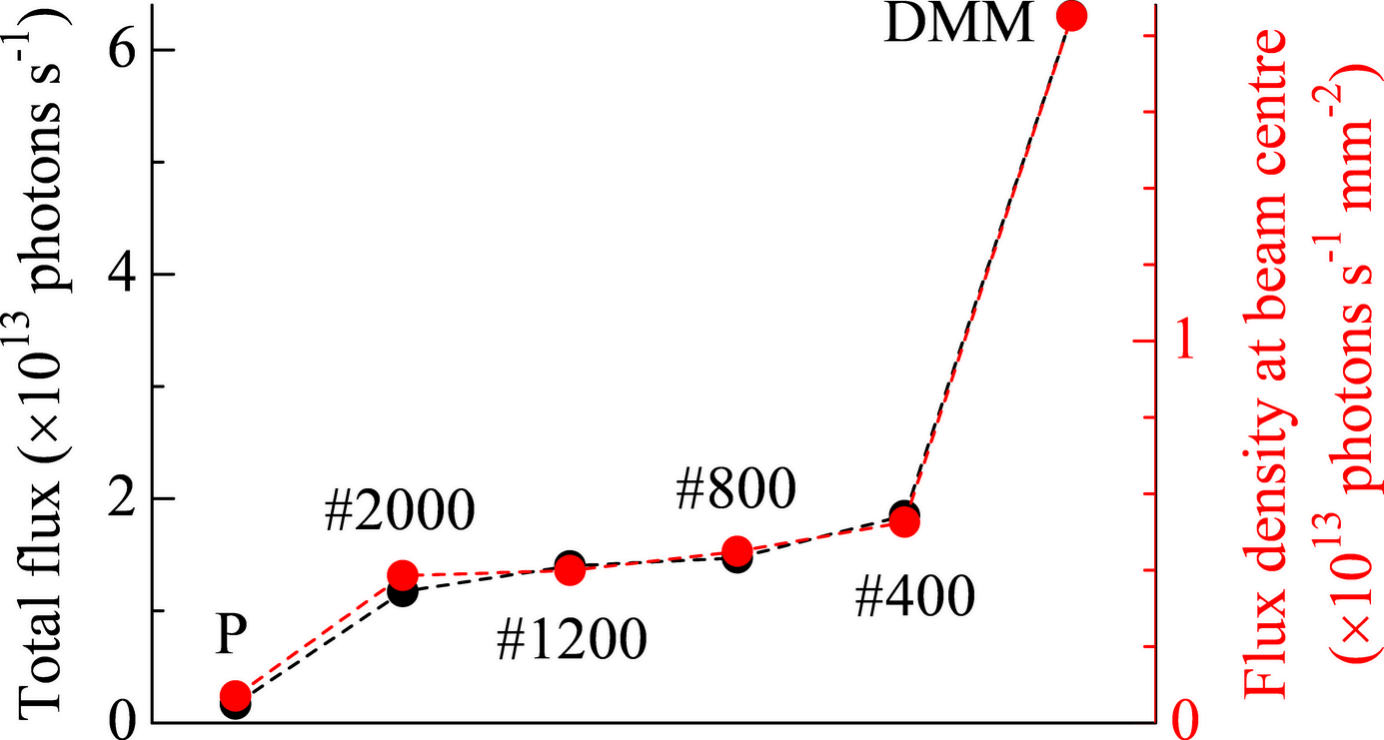
Comparison of total photon fluxes (black points) and flux densities at the beam centers measured through a 0.2 mm × 0.2 mm aperture (red points). Results are shown for the polished crystals, the lapped crystals (#2000–#400), and the DMM.

Fig. 5 ▸ summarizes the total photon fluxes for each crystal condition, together with the value obtained using the DMM. The #2000 and #400 lapped crystals delivered total fluxes of 1.2 × 10^13^ and 1.8 × 10^13^ photons s^−1^, corresponding to gains of approximately 7.0 and 11 relative to the polished crystals (0.17 × 10^13^ photons s^−1^). The flux from the #400 crystals was about one-third of that obtained from the DMM (6.3 × 10^13^ photons s^−1^).

The FWHMs of the rocking curves were 80 µrad (#2000) and 152 µrad (#400), in contrast to 4.6 µrad for the polished crystals. Although the measured profile for the polished crystals exhibited fluctuations associated with cryogenic cooling, the broader rocking-curve widths of the lapped crystals effectively reduced the impact of such instabilities, resulting in improved intensity stability.

#### 3.1.2. Beam profile imaging

**Figure 6:**
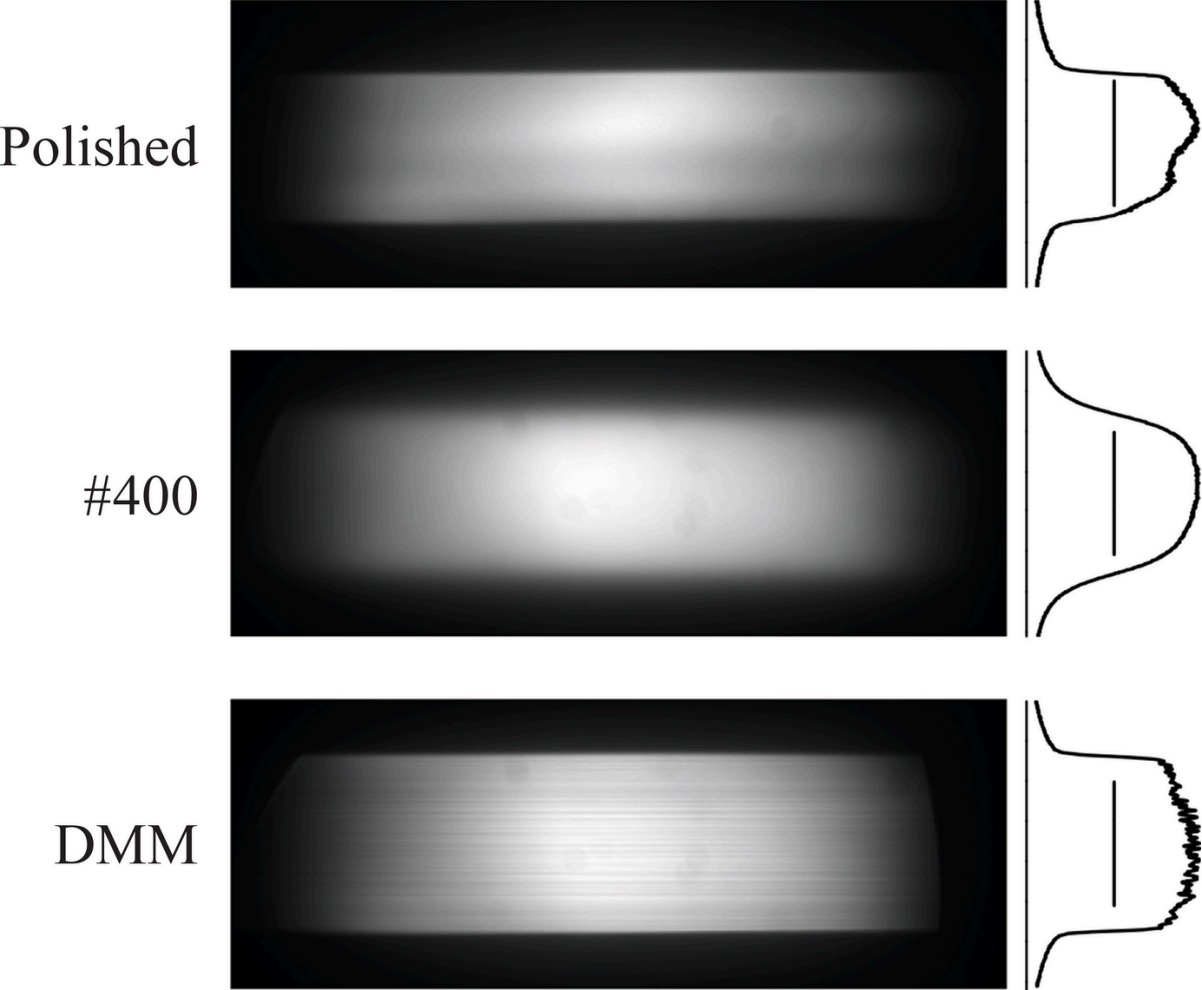
Beam images and corresponding vertical intensity profiles at the beam centers. Results are shown for the polished crystals, the #400 crystals, and the DMM. The intensity scale is normalized independently for each image. The polished crystals exhibit slow vertical modulation; the #400 crystals produce a uniform profile; and the DMM beam displays stripe patterns from multilayer figure errors. The bars represent 1 mm.

Fig. 6 ▸ presents beam images obtained using the polished crystals (exposure time: 200 ms), the #400 crystals (250 ms), and the DMM (100 ms). The intensity from the polished crystals was the lowest; however, the exposure time was reduced to mitigate vibration-induced blur. Intensity scale is normalized independently for each image. No noticeable differences were observed among the lapped crystals. The beam height (FWHM) increased in the order polished < #400 < multilayer.

The beam from the polished crystals exhibited slowly varying vertical intensity modulation, likely arising from sensitivity to clamping and thermal strain due to its narrow angular acceptance. In contrast, the #400 crystals produced a highly uniform beam profile. The DMM beam displayed fine stripe patterns, attributed to figure errors in the multilayer optics (Shimizu *et al.*, 2026 ▸). Faint circular structures (∼0.2 mm diameter) originated from the beam monitor.

#### 3.1.3. Wavelength–angle distribution

**Figure 7:**
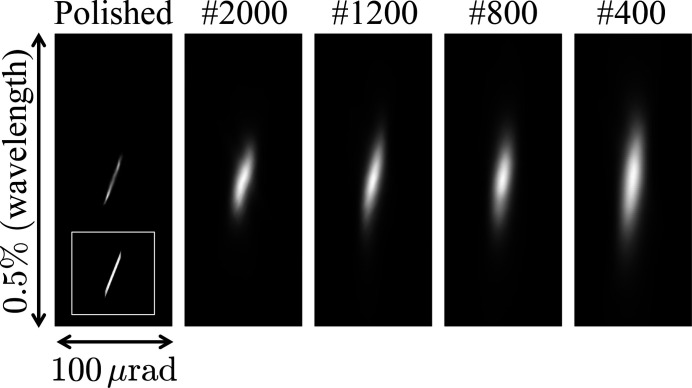
Reconstructed wavelength–angle distributions for each crystal condition. Vertical and horizontal full spans correspond to 0.5% in wavelength and 100 µrad in angle, respectively. Intensity scale is normalized independently for each distribution. For the polished crystals, the DuMond-predicted distribution (white frame) is shown for comparison.

Reconstructed wavelength–angle distributions at the peak rocking-curve positions are shown in Fig. 7 ▸. The shaded rectangles represent the distributions for each crystal condition. For the polished crystals, the distribution predicted by the DuMond analysis (DuMond, 1937 ▸) is also shown and agrees well with the reconstructed result, confirming the reliability of the reconstruction method.

**Figure 8:**
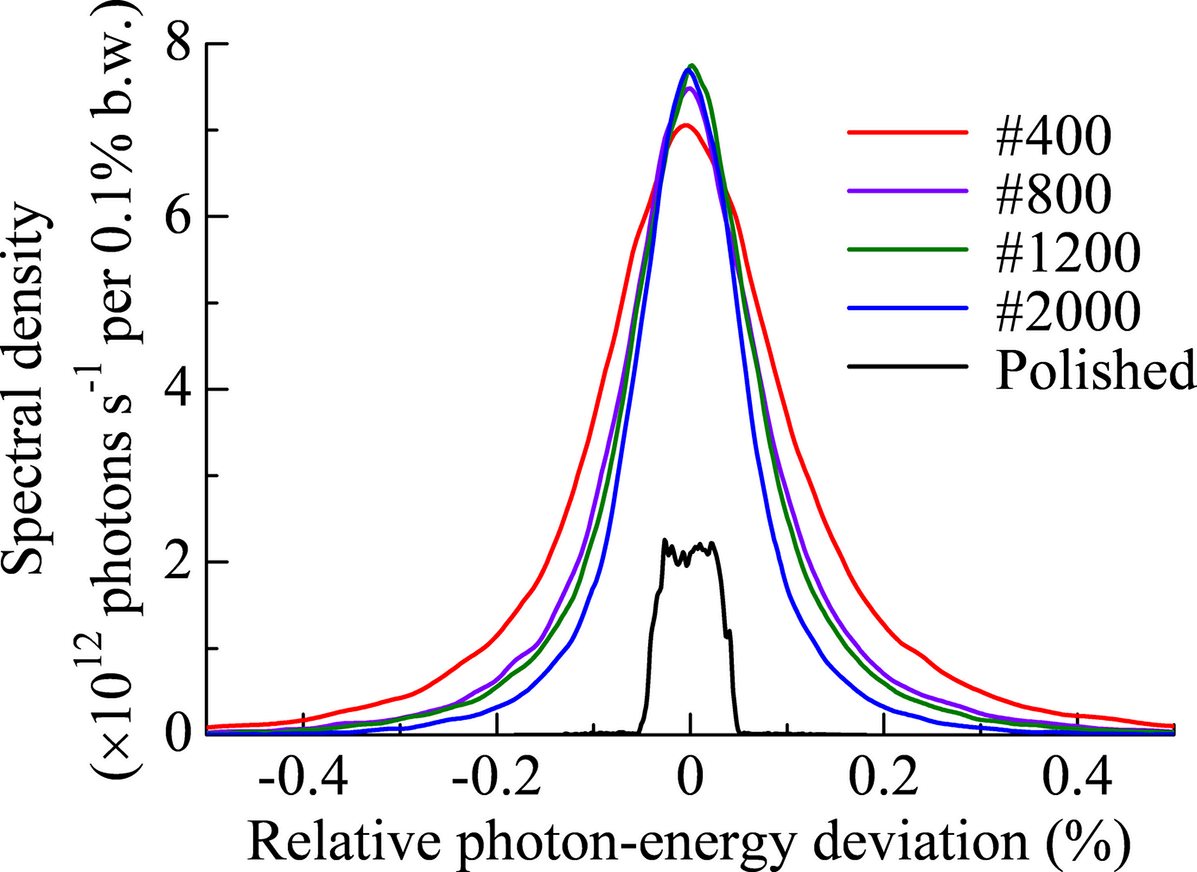
Energy spectra of the monochromated beams. The horizontal axis represents the relative photon-energy deviation (equal in magnitude to the inverse sign of the relative wavelength deviation). Spectral intensities are normalized by the peak flux of each rocking curve.

With increasing surface roughness, the wavelength spread broadened. Fig. 8 ▸ shows the energy spectra obtained by integrating the wavelength-angle distributions along the angular axis and normalizing by the peak flux of each rocking curve.

The angular width of the beam from the polished crystals was restricted by the angular divergence of the 100 keV component. The beams from the lapped crystals exhibited angular divergences of 20–24 µrad, which were close to the acceptance angle of the incident slit (0.56 mm/29 m ≃ 19 µrad).

#### 3.1.4. Flux density

The flux densities at the beam centers are plotted in Fig. 5 ▸. The lapped crystals increased the flux density by factors of 5.5–7.5 relative to the polished crystals, consistent with the trends observed in the total flux.

### 3.2. Applicable demonstrations

Only the results obtained using the #400 crystals are presented in this section. Although these crystals provided the highest flux, they also exhibited the broadest bandwidth and angular divergence, yielding the least favorable performance among the lapped crystals in the subsequent demonstrations.

#### 3.2.1. Transmission imaging

**Figure 9:**
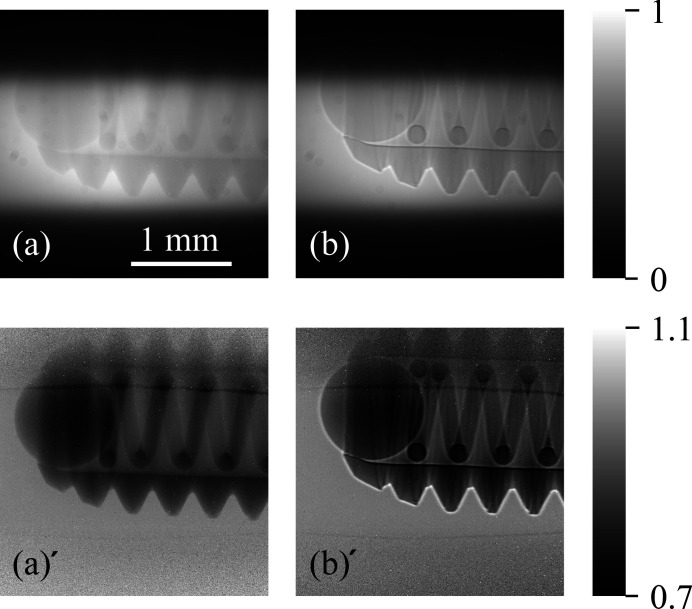
Transmission imaging of a spring-ball plunger using the #400 crystals. (*a*) Transmission image after 6 mm propagation; (*a*)′ normalized image of (*a*) after flat-field correction. (*b*) Transmission image after 2 m propagation; (*b*)′ normalized image of (*b*) after flat-field correction. The scale bar represents 1 mm.

Fig. 9 ▸(*a*) shows a transmission image of a miniature spring-ball plunger positioned 6 mm upstream of the beam imaging monitor, and Fig. 9 ▸(*a*)′ is the normalized image of Fig. 9 ▸(*a*) after flat-field correction. Figs. 9 ▸(*b*) and 9 ▸(*b*)′ were taken when the plunger was positioned 2 m upstream of the monitor. The increase in contrast was clearly observed after the 2 m propagation. The exposure times were 250 ms for both cases. Impulse noise originated from the detector.

#### 3.2.2. Powder diffraction

**Figure 10:**
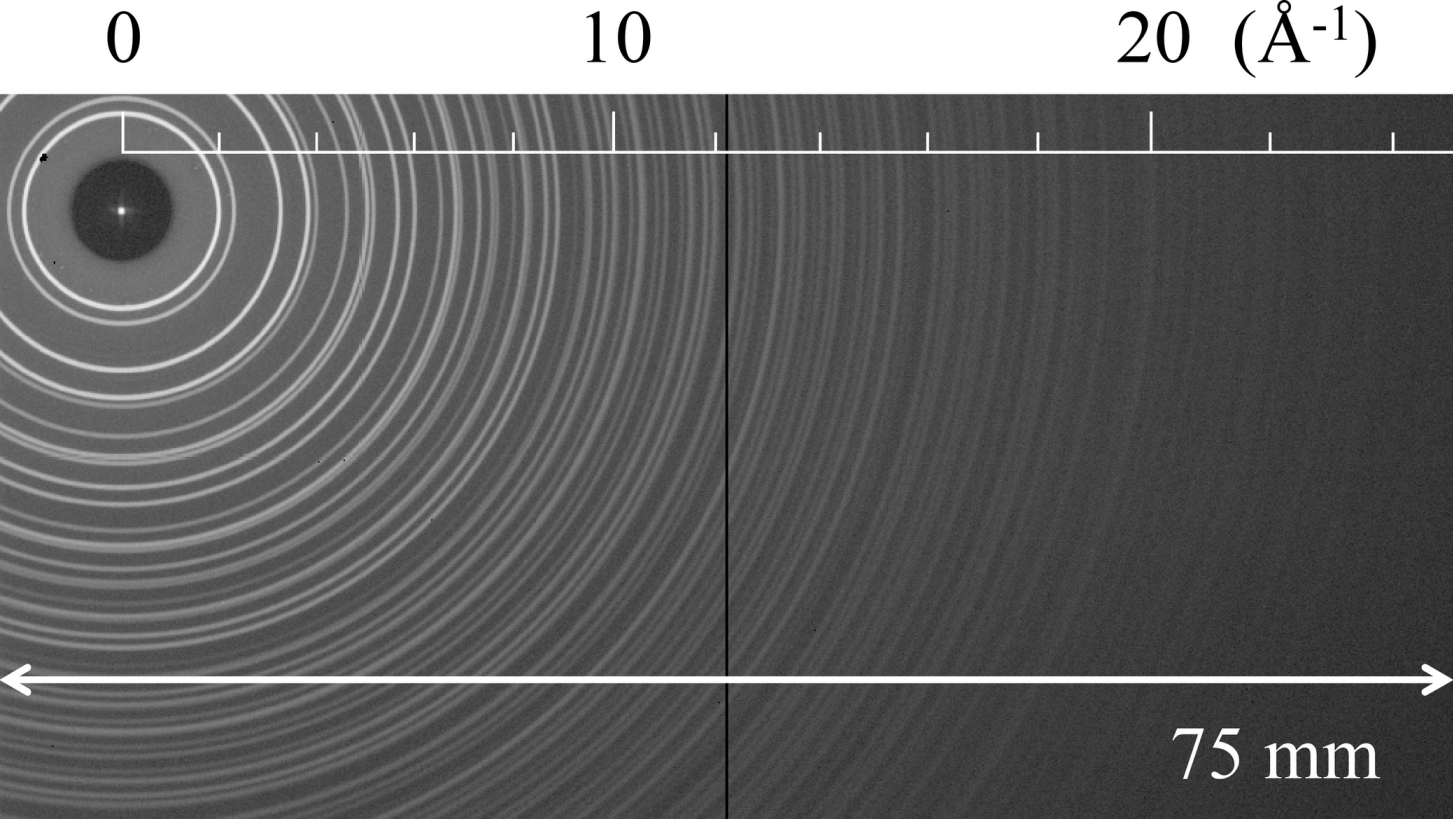
Debye–Scherrer rings from CeO_2_ powder measured using the #400 crystals. The displayed area corresponds to half of the detector area. The indicator shows the scattering-vector magnitude. A logarithmic (log_10_) intensity scale is used to enhance visibility of peripheral rings.

Fig. 10 ▸ displays Debye–Scherrer rings from CeO_2_ powder. A 1 s exposure was sufficient to record diffraction peaks up to a scattering-vector magnitude of *q* = 25 Å^−1^. A logarithmic (log_10_) intensity scale was applied to enhance the visibility of the peripheral rings. The intense central spot corresponds to the transmitted beam, followed by the shadow of the beam stopper.

#### 3.2.3. Focusing with KB mirror

The incident flux into the KB mirror was 4.6 × 10^11^ photons s^−1^ within a 0.30 mm × 0.28 mm cross section. Knife-edge scans yielded focal spot sizes of 4.5 µm (FWHM; vertical) and 6.3 µm (horizontal), with a flux of 3.4 × 10^11^ photons s^−1^, corresponding to more than a 2000-fold increase in flux density.

For comparison, the DMM beam focused with the same mirror achieved focal sizes of 0.32 µm (vertical) and 5.3 µm (horizontal) at an intensity of 1 × 10^12^ photons s^−1^, corresponding to the high-flux mode defined by Koyama *et al.* (2024 ▸).

The broadening of the vertical focal size for the beam from the #400 crystals is most likely caused by diffusion effects on the lapped crystals, reflecting a degradation of the wavefront quality.

## 4. Discussion

Compared with a conventional DCM employing polished Si(111) crystals, lapping the crystal surfaces substantially enhanced the performance of high-energy X-rays. For the #400 crystals, the total flux and flux density increased by factors of up to 11 and 7.5, respectively, while the resulting energy bandwidth remained at 0.21%, which is suitable for standard diffraction experiments. The resulting beam from these crystals was also compatible with standard imaging experiments owing to its uniformity. The angular divergence of 24 µrad, being close to the acceptance angle of the incident slit (19 µrad), did not compromise the demonstrated propagation-based edge-contrast imaging under the present experimental conditions.

The broadening of the rocking-curve profiles remarkably reduces the sensitivity to intensity fluctuations caused by angular vibrations within the DCM system. In addition, the beam-profile measurements indicate that lapping effectively mitigates spatial non-uniformities arising from clamping or thermal strain—issues that inherently affect polished crystals due to their narrow angular acceptance.

Compared with the DMM, the lapped crystals delivered a total flux approximately one-third as high. However, the lapped crystals provided beams free from low-energy contamination and thus required no low-energy-cut filter, unlike high-energy multilayer optics where shallow glancing angles lead to low-energy contamination via total reflection. The lapped crystals also offer two practical advantages over multilayers: they can be fabricated at considerably lower cost, and their compact volume facilitates mounting on precision rotation and translation stages.

Overall, lapped Si crystals represent an attractive solution for generating high-flux, moderate-bandwidth, and spectrally pure X-ray beams in high-energy regions. Their properties—including moderate angular divergence, intensity stability against mechanical vibration, spatial uniformity, and cost-effectiveness—motivate the further development of energy-tunable DCM systems for high-energy X-rays. Extending this approach to a wider range of photon energies and optimizing lapping conditions to tailor the wavelength–angle distributions for specific applications represent important directions for future work.

While the present study demonstrates that lapped Si(111) crystals provide high-flux X-ray beams with practically useful moderate bandwidths for diffraction and imaging applications, a more detailed quantitative description of the wavelength–angle correlations requires a dedicated analysis beyond the scope of this work. Such analyses are essential for rigorously defining and comparing moderate-bandwidth beams and will be addressed separately (Yamazaki *et al.*, 2026 ▸).

## 5. Conclusion

Lapping the reflecting surfaces of Si(111) crystals, exemplified here by 100 keV X-rays, increased the total beam flux by factors of 7–11 and the flux density by 5.5–7.5, depending on the surface roughness. The resulting beams from the lapped crystals exhibited energy bandwidths of 0.12–0.21%, which are suitable for diffraction experiments. Unlike the DMM, lapped crystals produce no low-energy contamination. The moderate angular divergences of 20–24 µrad and the highly uniform spatial profiles indicate that the beams are also well suited for imaging applications, including the edge-contrast imaging demonstrated here.

Lapped crystals further provide practical advantages arising from their broadened rocking-curve widths, which render the DCM more robust against intensity fluctuations caused by mechanical vibrations. Their compact size also facilitates mounting on precision alignment stages. These characteristics motivate continued development of high-flux, energy-tunable DCM systems for high-energy X-rays and suggest that further optimization of lapping parameters could enable tailoring of wavelength–angle distributions for specific experimental needs.

## Figures and Tables

**Table 1:** Summary of the main beam parameters obtained using the polished and the lapped crystals, and the DMM

	Polished	#2000	#1200	#800	#400	DMM
Total flux (×10^13^ photons s^−1^)	0.17	1.2	1.4	1.5	1.8	6.3
Rocking-curve width (µrad)	4.6	80	102	116	152	–
Energy bandwidth (%)†	0.08	0.12	0.14	0.16	0.21	1.0‡
Angular divergence (µrad)†	13	21	20	21	24	–
Flux density (×10^13^ photons s^−1^ mm^−2^)§	0.07	0.39	0.40	0.45	0.53	1.9

† FWHM values were calculated from the reconstructed wavelength–angle distributions (see §3.1.3).

‡ DMM values are taken from Yumoto *et al.* (2025 ▸).

§ Flux densities were defined through a 0.2 mm × 0.2 mm aperture.

## Data Availability

The data supporting this study are available from the corresponding author upon reasonable request.
